# A novel ultrasound scanning approach for evaluating femoral cartilage defects of the knee: comparison with routine magnetic resonance imaging

**DOI:** 10.1186/s13018-018-0887-x

**Published:** 2018-07-16

**Authors:** Junyan Cao, Bowen Zheng, Xiaochun Meng, Yan Lv, Huading Lu, Kun Wang, Dongmei Huang, Jie Ren

**Affiliations:** 1 Department of Medical Ultrasonics, The Third Affiliated Hospital of Sun Yat-sen University, 600 Tianhe Road, Guangzhou, 510630 People’s Republic of China; 2 Department of Radiology, The Third Affiliated Hospital of Sun Yat-sen University, 600 Tianhe Road, Guangzhou, 510630 People’s Republic of China; 3 Department of Orthopedics, The Third Affiliated Hospital of Sun Yat-sen University, 600 Tianhe Road, Guangzhou, 510630 People’s Republic of China

**Keywords:** Cartilage disease, Ultrasound, Magnetic resonance imaging, Diagnostic performance, Arthroscopy, Sensitivity, Specificity

## Abstract

**Background:**

This study aimed to assess a novel ultrasound (US) scanning approach in evaluating knee femoral cartilaginous defects, compared with magnetic resonance imaging (MRI, commonly used for knee imaging) and arthroscopy (gold standard).

**Methods:**

Sixty-four consecutive patients (65 knees) were prospectively evaluated between April 2010 and July 2011.

**Results:**

The overall sensitivity (62.2 and 69.4%), specificity (92.9 and 90.5%), accuracy (75.4 and 78.5%), and adjusted positive (88.7 and 90.4%) and negative predictive (69.5 and 73.3%) were similar for both radiologists (weighted *κ* = 0.76). Furthermore, agreement between grading by US and MRI was substantial (weighted *κ* = 0.61).

**Conclusions:**

In conclusion, the novel US scanning approach allows similar diagnostic performance compared to routine MRI for knee cartilage defects. US is more accessible, easier to perform, and less expensive than MRI, with potential advantages of easier initial screening and assessment of cartilage defects.

**Electronic supplementary material:**

The online version of this article (10.1186/s13018-018-0887-x) contains supplementary material, which is available to authorized users.

## Background

Progressive articular cartilage defects in the knee are a major cause of pain, disability, and medical expenses in the general population and particularly in the elderly [1]. Precise assessment of cartilaginous abnormality is important to determine the appropriate treatments, e.g., osteotomy, mosaicplasty, drugs, and autologous chondrocyte transplantation [2]. Ultrasound (US) is widely available and relatively inexpensive and has proven to be a useful modality for the routine clinical assessment of joint diseases [3]. US is radiation-free and non-invasive and allows dynamic assessment of moving structures [4]. Previous studies evaluated the feasibility and diagnostic value of US for detecting cartilaginous defects [5–15]. Previous studies reported associations of US with histologic and arthroscopic classifications of cartilage defects [11, 13], and with an acceptable diagnostic performance [14], suggesting that knee US is a promising technique for screening degenerative changes of articular cartilage in patients with osteoarthritis (OA) [14]. Nevertheless, the correlations observed in previous studies were low, with coefficients of 0.262–0.655 [13, 14] and could be due to the selection of the indicators, the selection of US systems, and the experience of the raters. In addition, negative predictive values (NPV) remained low (23.8–45.8%), implying that a negative finding using US does not rule out cartilage degenerative changes [14]. Although a positive finding in US is a strong indicator of arthroscopic degenerative changes of cartilage [14], the technique still needs improvements before routine clinical use.

Previous assessments were performed with a transducer capable of the highest frequency available for routine clinical use, already resulting in the highest sensitivity possible [13, 14]. The diagnostic accuracy of US is often dependent upon the scanning approach in different US indications [16–18]. Therefore, we hypothesized that improvement of the scanning technique could enhance US diagnostic value. Indeed, previous studies used a fixed flexed knee (e.g., 120°) with transverse scanning only [13, 14], but the knee flexion angle influences the correlation between US and histologic classification of cartilage defects, although the optimal angle remains unknown [13]. In addition, since only transverse scanning was applied, it remains unclear whether longitudinal scanning could offer more benefits, especially when depicting the condyle [14].

Therefore, this study aimed to assess a novel US scanning approach in evaluating knee femoral cartilaginous defects, compared with magnetic resonance imaging (MRI, commonly used for knee imaging) and arthroscopy (gold standard).

## Methods

### Study design and patients

In this prospective study, 64 consecutive patients (65 knees) scheduled for knee arthroscopy between April 2010 and July 2011 were prospectively evaluated by US and MRI at our hospital. The study was approved by the research ethics committee of the Third Affiliated Hospital of Sun Yat-sen University. All patients signed an informed consent prior to participation in the study.

Patients with a chief complaint of knee pain or disability and scheduled for arthroscopy were enrolled in this study. The patients underwent arthroscopy of the knee because of suspicion of internal derangement. Patients with prior knee surgery (e.g., total knee arthroplasty) and contraindications to MRI were excluded.

### US examination

**Fig. 1:**
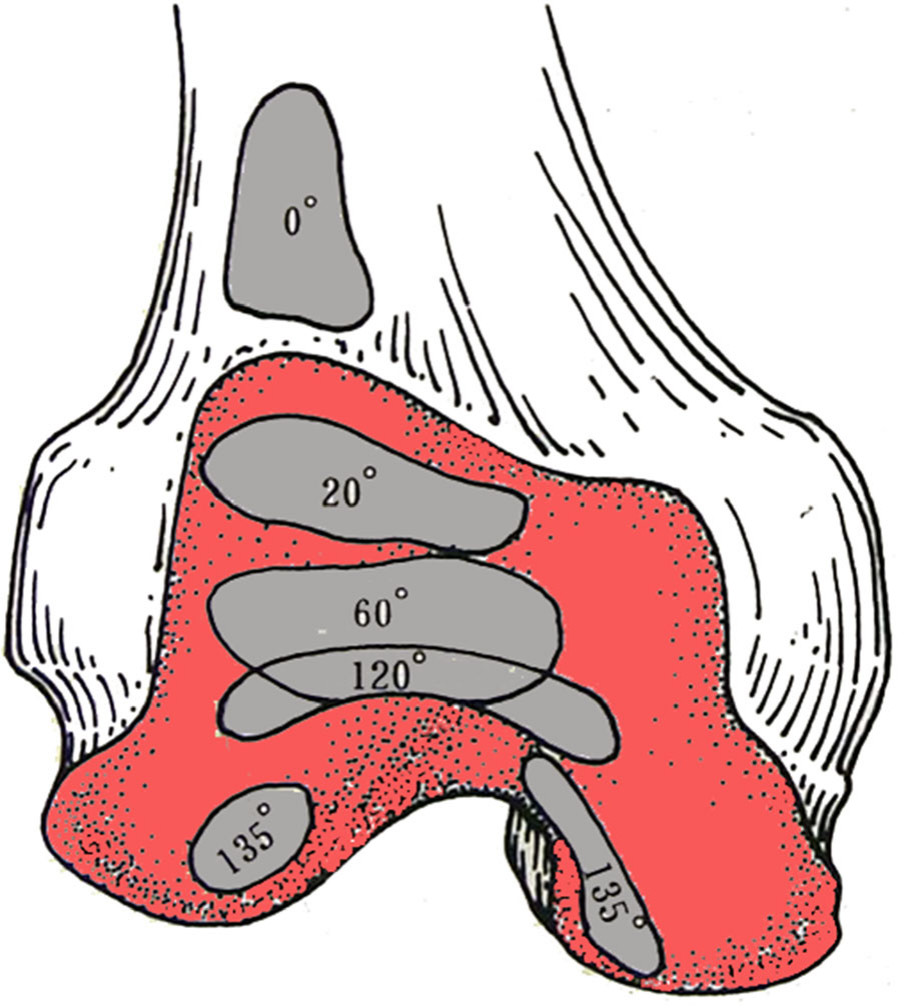
Contact areas of the patellofemoral articulation during different arcs of motion, adapted from Shahriaree’s O’Connor’s Textbook of Arthroscopic Surgery [15]. Red area, femoral cartilage; gray areas, contact areas of the femoral cartilage of the patellofemoral articulation during different arcs of motion

**Fig. 2:**
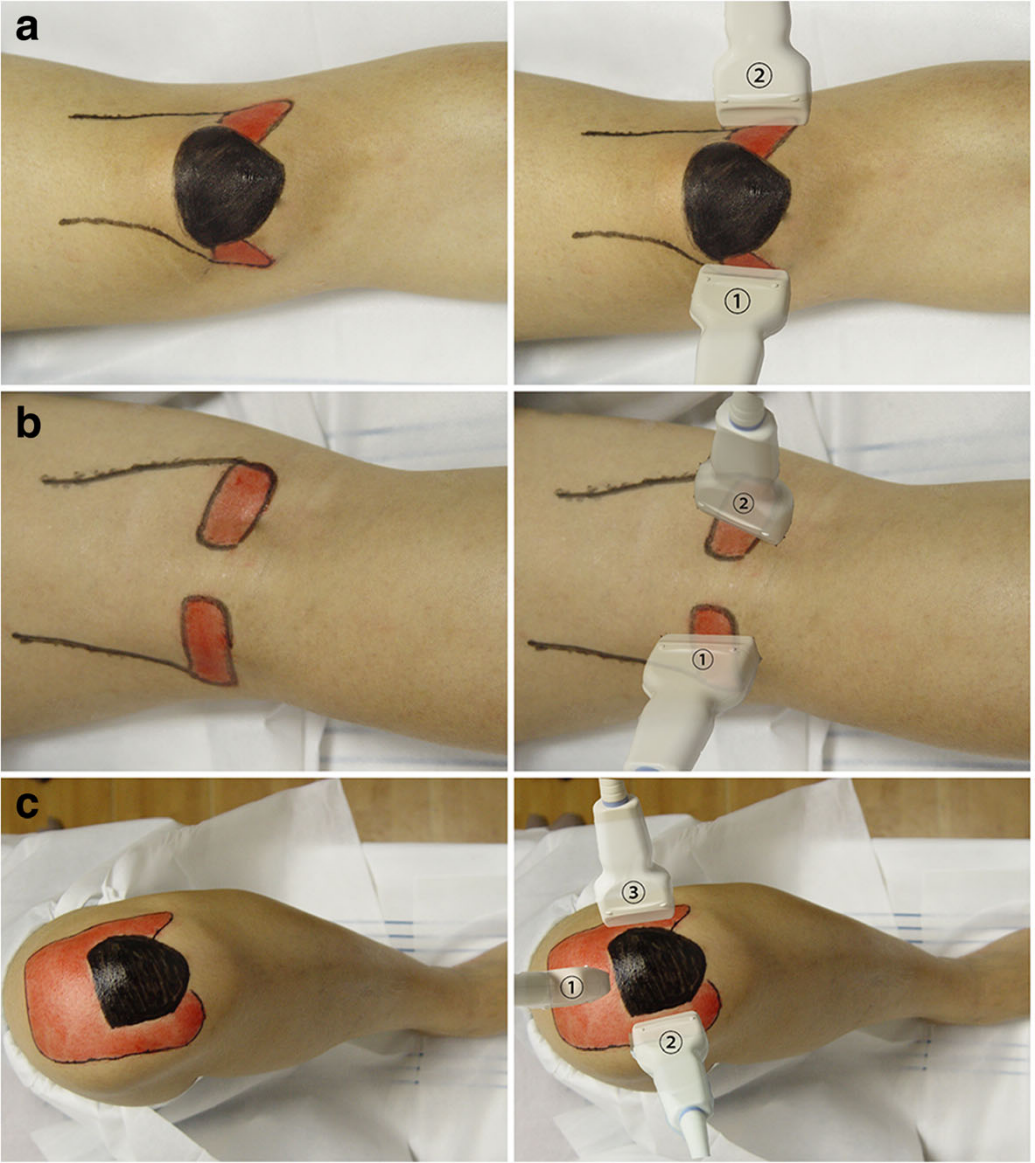
Novel ultrasound (US) scanning approach for the detection of femoral cartilage. **a** With fully extended knee (0°), transverse and longitudinal scanning of bilateral sides of the patella for cartilage of the anterior portion of medial (1) and lateral (2) condyles with the patient in the supine position. **b** Transverse and longitudinal scanning of the popliteal fossa for the posterior portion of medial (1) and lateral (2) condyles with the patient in a prone position with a fully extended knee. **c** With maximum knee flexion in the supine position, transverse and longitudinal scanning of the trochlear surface (1) and weight-bearing portion of medial (2) and lateral (3) condyles. Red area, surface projection of the femoral cartilage; black area, surface projection of the patella

US examination of the knee joint was conducted a week before arthroscopy using a LOGIQ 700 (GE Medical Systems, Milwaukee, WI, USA) with a 7–9-MHz high-resolution linear transducer. A novel US scanning approach based on the functional anatomy of the knee (Fig. 1) [15] was proposed, considering articular motions and both transverse and longitudinal scanning (Fig. 2). (1) In the supine position with a fully extended knee (0°), transverse and longitudinal scanning of bilateral sides of the patella for the anterior portions of both condyles was carried out (Fig. 2a). (2) In the prone position, longitudinal and transverse scanning of the popliteal fossa for the posterior portions of both condyles was performed (Fig. 2b). (3) In the supine position with maximum knee flexion, transverse and longitudinal scanning of the suprapatellar recess for the trochlear surface was undertaken (Fig. 2c). (4) Finally, in the same position, transverse and longitudinal scanning of bilateral sides of the patella was performed again, for the weight-bearing portion of the condyles (Fig. 2c). The US beam was always kept perpendicular to the femur surface. Maximum flexion angle of each patient was recorded, and an angle > 135° was considered good flexion. This angle was confirmed using a lateral photography and the Angler app (an iOS app for angle measurement which was available only on Apple Store China but is unfortunately no longer available). The patient was in the supine position with maximum knee flexion, then a lateral photography was taken and the flexion angle was measured by this app.

A US grading system for knee femoral cartilage defects, based on the International Cartilage Repair Society (ICRS) classification [5, 7, 19, 20], was used: grade 0, normal cartilage; grade 1, nearly normal cartilage with blurred margin or partial lack of clarity without thickness change; grade 2, abnormal cartilage with blurring or obliteration of the margin, lack of clarity, and overt local thinning of the cartilage (< 50% of cartilage depth); grade 3, severely abnormal cartilage with blurring and obliteration of the margin and obvious focal thinning for > 50% of the cartilage depth but intact cartilage-bone interface; and grade 4, severely abnormal cartilage with complete loss of cartilage and coarse or irregular cartilage-bone interface.

Two musculoskeletal radiologists (JYC and JR, with 5 and 15 years of clinical experience, respectively) were trained for 2 months to perform the novel examination method before the present study. This training involved ten volunteers. The radiologists repeatedly scanned those volunteers, alone and together, and compared their results. In this study, the radiologists scanned each patient immediately one after another (according to their availability, without a predefined order) and made assessments independently during real-time scanning, based on individual findings. A form with a schematic drawing of the articular surfaces of the femoral cartilage [19] was used. The trochlear surface and lateral and medial femoral condyles were evaluated separately. In the presence of multiple cartilaginous defects on the same articular surface, the worst score was attributed.

### MRI evaluation

MRI examinations were performed within 1 week before arthroscopy on a Signa Excite 1.5 Tesla MR System (GE Medical Systems, Milwaukee, WI, USA), using a phased array knee coil. Patients were placed in the supine position with the knee fully extended. All MRI examinations consisted of sagittal T1- and T2-weighted, sagittal fat-saturated (fs) proton density (PD)-weighted, coronal fs PD-weighted, and axial fs PD-weighted fast spin echo (FSE) sequences. The imaging parameters are presented in Additional file 1: Table S1.

All MRI images were interpreted by one radiologist (XCM) with over 15 years of experience in musculoskeletal imaging. The radiologist was blinded to the clinical history and US findings. The cartilage was morphologically graded according to a modified ICRS classification system [19]: grade 0, normal cartilage; grade 1, cartilage with intact surface and no tissue loss, but fibrillation and superficial fissures; grade 2, deep defects, but < 50%; grade 3, lesions representing > 50% of the cartilage thickness; and grade 4, defects extending into the subchondral bone. The same schematic drawing of articular surfaces of the femoral cartilage was used to mark defect locations and degrees. The trochlear surface and lateral and medial femoral condyles were evaluated separately. In the presence of multiple cartilaginous defects on the same articular surface, the worst score was attributed.

### Arthroscopy

Two orthopedic surgeons (HDL and KW, with 15 and 25 years of clinical experience, respectively) were involved in this study. They were blinded to the US and MRI findings. Knee arthroscopy with fluid irrigation was performed using the standard anterolateral and anteromedial portals to assess the articular cartilage defects and potential comorbidities. The surgeons were free to flex and extend the patients’ knees. For cartilage evaluation by arthroscopy, the same schematic drawing of the femoral cartilage surfaces of the knee was used. The surgeons performed their assessment in consensus. The trochlear surface and lateral and medial femoral condyles were evaluated separately. The cartilage lesions were graded analogously to the US and MRI using the ICRS classification [19]. In the presence of multiple cartilaginous defects on the same articular surface, the worst score was attributed. Arthroscopic grading served as the gold standard.

### Statistical analysis

With arthroscopic findings as a reference, sensitivity, specificity, and accuracy of US and MRI for femoral cartilage defects were assessed. In addition, positive predictive value (PPV) and NPV were calculated and adjusted for the prevalence of femoral cartilage lesions documented in arthroscopy at our institution (51.9%), based on previous recommendations [21]. The threshold of femoral cartilage abnormality was set between grade 0 as negative and grades 1–4 as positive. Furthermore, detection rates of each grade of cartilage defects and proportion of cartilage lesions graded identically and within one grade in arthroscopy and imaging modalities were calculated. The McNemar chi-square test was used to compare the groups.

Weighted *κ* statistics for the level of agreement among different methods and between radiologists (US) were used [22]. The Student *t* test was used to compare the *κ* values between grades assigned by arthroscopy and US or MRI. Error analysis was performed for the first blinded US evaluation by the same radiologists after the revelation of arthroscopic findings. All false-positive or false-negative results were analyzed, and the most likely reason for the diagnostic error was recorded.

All statistical analyses were performed with SPSS 17.0 (IBM, Armonk, NY, USA) and STATA 11 (StataCorp LP, College Station, TX, USA). Two-sided *P* values < 0.05 were considered significantly significant.

## Results

### Patient baseline characteristics and arthroscopy data

Sixty-four consecutive patients (65 knees) with various abnormalities of the knee were assessed, including 21 men and 43 women, with a mean age of 42 years (range, 18–75). Final diagnoses included OA (25 knees), meniscus injury (11 knees), traumatic arthritis (11 knees), chondromalacia patella (7 knees), anterior cruciate ligament tears (6 knees), dislocation of the patella (3 knees), and meniscus cyst (2 knees). Among the 65 knees, 90.8% (59/65) had a good flexion. Altogether, 195 knee femoral cartilage surfaces, including the trochlear surface (TS), lateral femoral condyles (LC), and medial femoral condyles (MC), were evaluated by US, MRI, and arthroscopy. Arthroscopy revealed that 84, 20, 30, 38, and 23 surfaces had grades 0, 1, 2, 3, and 4 defects, respectively (Table 1). The distribution of cartilage defects identified by arthroscopy is shown in Table 1.

**Table 1:** Location and grades of cartilaginous defects in 65 knees by arthroscopy

Surface	Grade 0	Grade 1	Grade 2	Grade 3	Grade 4
TS	27 (13.9)	7 (3.6)	11 (5.6)	13 (6.7)	7 (3.6)
MC	25 (12.8)	7 (3.6)	11 (5.6)	14 (7.2)	8 (4.1)
LC	32 (16.4)	6 (3.1)	8 (4.1)	11 (5.6)	8 (4.1)
Total	84 (43.1)	20 (10.3)	30 (15.3)	38 (19.5)	23 (11.8)

Data are the numbers of surfaces. Values in parentheses are percentages

*TS* trochlear surface, *MC* medial condyles, *LC* lateral condyles

### Overall diagnosis performances of US and MR

**Fig. 3:**
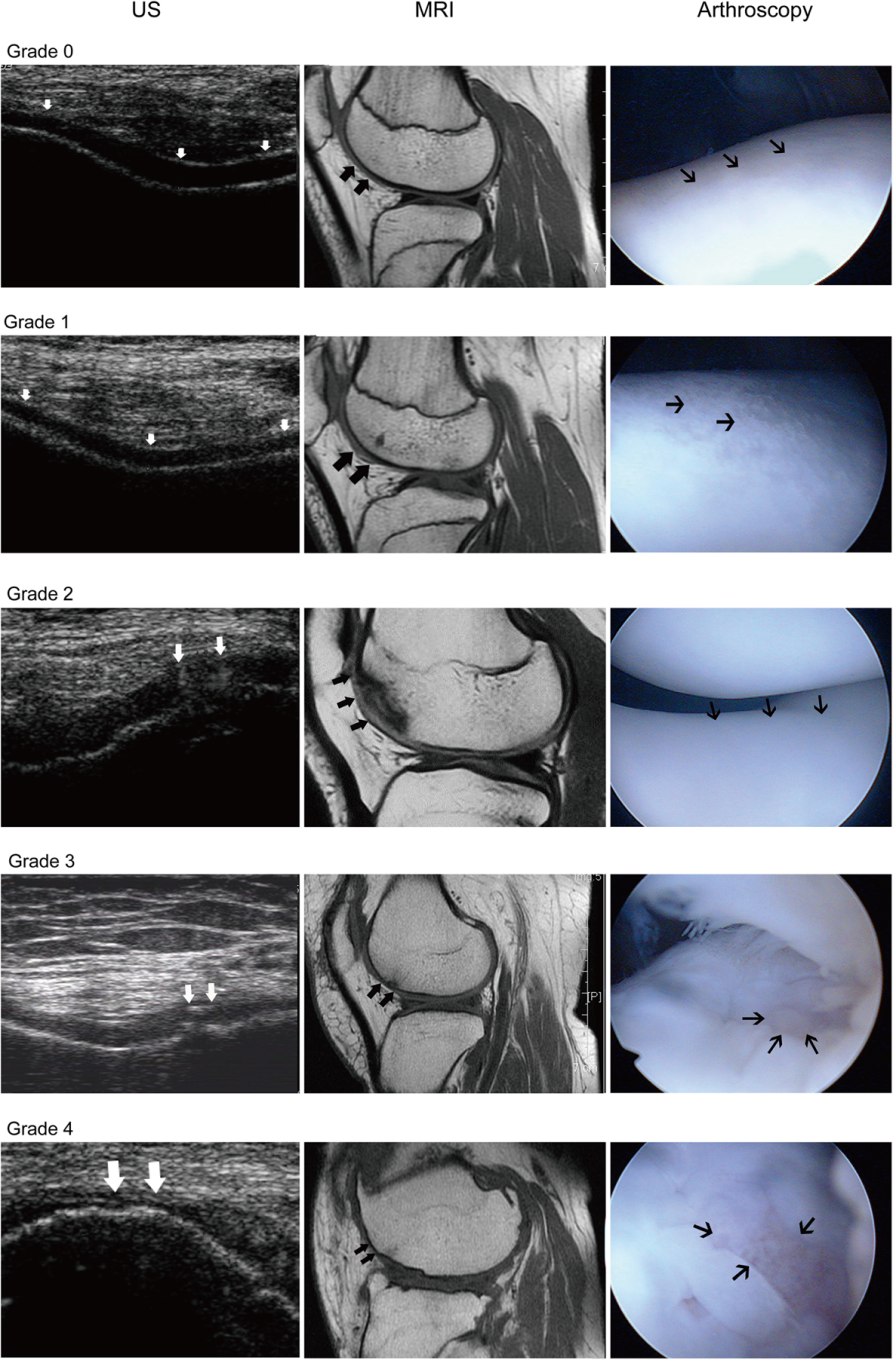
Typical cases of each grade of cartilage defects at the trochlear surface. Defects are shown as white arrows for US, bold black arrows for MRI, and thin black arrows for AS

Table 2 shows the overall sensitivity, specificity, accuracy, and crude and adjusted PPV and NPV for US and MRI in detecting knee femoral cartilage defects, using arthroscopy as the gold standard. Figures 3 presents typical cases of each grade of cartilage defects. The sensitivity (62.2 and 69.4%), specificity (92.9 and 90.5%), and accuracy (75.4 and 78.5%) were similar between two independent radiologists performing the US. Interobserver agreement was obtained between the two radiologists, as indicated by a weighted *κ* value of 0.76. Considering the prevalence of cartilage lesions of 51.9% at our institution, obtained based on the analysis of 832 knee arthroscopies from January 2002 to December 2011, adjusted PPV was 88.7–90.4% (false PPV = 9.6–11.3%), and adjusted NPV was 69.5–73.3% (false NPV = 26.7–30.5%). Only a lower sensitivity of US was observed for radiologist 1 (JYC, 5 years of clinical experience) compared with MRI (*P* = 0.042). Other parameters showed non-significant differences between US and MRI (*P* = 0.060 to 1.000), as shown in Table 2.

**Table 2:** Overall diagnostic performance and predictive value of US and MRI in evaluating cartilaginous defects

		TP^#^	TN^#^	FP^#^	FN^#^	Sensitivity (95%CI) (%)	Specificity (95%CI) (%)	Accuracy (95%CI) (%)	Crude predictive value (95%CI) (%)		Adjusted predictive value (%)		AUC (95% CI)
									PPV	NPV	PPV	NPV	
US	Radiologist 1	69	78	6	42	62.2 (53.1–71.2) (0.042)	92.9 (87.3–98.4) (1.000)	75.4 (69.3–81.4) (0.060)	92.0 (85.9–98.1) (0.756)	65.0 (56.5–73.5) (0.148)	90.4	69.5	0.775 (0.722–0.828)
	Radiologist 2	77	76	8	34	69.4 (60.8–77.9) (0.367)	90.5 (84.2–96.8) (0.565)	78.5 (72.7–84.2) (0.245)	90.6 (84.4–96.8) (0.397)	69.1 (60.5–77.7) (0.450)	88.7	73.3	0.799 (0.746–0.853)
MRI		84	79	5	27	75.7 (67.7–83.7)	94.0 (89.0–99.1)	83.6 (78.4–88.8)	94.4 (89.6–99.2)	74.5 (66.2–82.8)	93.2	78.2	0.849 (0.801–0.896)

Numbers in parentheses are the *P* values compared with MRI data

*TP* true positive, *TN* true negative, *FP* false positive, *FN* false negative

^#^Data are the surface numbers

### US and MR diagnosis performance comparisons for different surfaces

For all cartilage defect grades on each articular surface, some differences were seen between US and MRI for specific surfaces (Table 3), but there were differences between the two radiologists.

**Table 3:** Diagnostic performance of US and MRI for evaluating cartilaginous defects on different surfaces

Surface	TP^#^	TN^#^	FP^#^	FN^#^	Sensitivity (95%CI) (%)	Specificity (95%CI) (%)	Accuracy (95%CI) (%)	Crude predictive value (95%CI) (%)		Adjusted predictive value (%)		AUC (95%CI)
								PPV	NPV	PPV	NPV	
US Radiologist 1												
TS	31	25	2	7	81.6 (69.3–93.9) (0.317)	92.6 (82.7–102) (1.000)	86.2 (77.8–94.6) (1.000)	93.9 (85.5–102) (1.000)	78.1 (63.8–92.4) (1.000)	92.2	82.3	0.871 (0.791–0.951)
MC	20	22	3	20	50.0 (34.5–65.5) (0.005)	88.0 (75.3–101) (0.046)	64.6 (53.0–76.2) (0.058)	87.0 (73.2–100) (0.310)	52.4 (37.3–67.5) (0.251)	81.8	62.0	0.690 (0.588–0.792)
LC	18	31	1	15	54.5 (37.6–71.5) (0.083)	96.9 (90.8–103) (0.317)	75.4 (64.9–85.9) (0.046)	94.7 (84.7–105) (1.000)	67.4 (53.8-80.9) (0.818)	95.0	66.4	0.757 (0.666–0.849)
US Radiologist 2												
TS	35	24	3	3	92.1 (83.5–101) (0.025)	88.9 (77.0–101) (0.317)	90.8 (83.7–97.8) (0.424)	92.1 (83.5–101) (1.000)	88.9 (77.0–101) (0.315)	90.0	91.3	0.905 (0.831–0.979)
MC	22	22	3	18	55.0 (39.6–70.4) (0.248)	88.0 (75.3–101) (0.014)	67.7 (56.3–79.1) (0.157)	88.0 (75.3–101) (0.424)	55.0 (39.6–70.4) (0.352)	83.2	64.4	0.715 (0.613–0.817)
LC	20	30	2	13	60.6 (43.9–77.3) (1.000)	93.8 (85.4–102) (0.317)	76.9 (66.7–87.2) (1.000)	90.9 (78.9–103) (0.424)	69.8 (56.0–83.5) (1.000)	91.3	68.8	0.772 (0.677–0.867)
MRI												
TS	30	25	2	8	78.9 (66.0–91.9)	92.6 (82.7–102)	84.6 (75.8–93.4)	93.8 (85.4–102)	75.8 (61.1–90.4)	92.0	80.3	0.858 (0.775–0.940)
MC	28	24	1	12	70.0 (55.8–84.2)	96.0 (88.3–104)	80.0 (70.3–89.7)	96.6 (89.9–103)	66.7 (51.3–82.1)	95.0	74.8	0.830 (0.748–0.712)
LC	21	30	2	12	63.6 (47.2–80.0)	93.8 (85.4–102)	78.5 (68.5–88.5)	91.3 (79.8–103)	71.4 (57.8–85.1)	91.7	70.5	0.787 (0.693–0.881)

Numbers in parentheses are the *P* values compared to MRI data (McNemar test)

*TS* trochlear surface, *MC* medial condyles, *LC* lateral condyles, *TP* true positive, *TN* true negative, *FP* false positive, *FN* false negative

^#^Data are the surface numbers

### US and MR diagnosis performance comparisons for different defect grades

The respective detection rates and comparisons between US and MRI for cartilage defects of each grade are shown in Tables 4 and 5. Generally, compared with MRI, no significant differences were obtained in detecting grades 0, 2, 3, and 4 defects (*P* = 0.083 to 0.317), but a lower detection rate with US for grade 1 defects was obtained for both radiologists (*P* = 0.025). Indeed, for radiologist 1, ten lesions were diagnosed as grade 0, eight as grade 2, and two as grade 3. For radiologist 2, eight lesions were diagnosed as grade 0, six as grade 2, and six as grade 3. The proportions of cartilage defects graded identically and matched within one grade of arthroscopic values using US by the two radiologists were 80.0 and 80.5%, respectively, with no significant difference between both radiologists (*P* = 0.179 and *P* = 0.224, respectively). A moderate agreement between arthroscopy and US for grade assignment (weighted *κ* = 0.60, 95% CI 0.50–0.71, and 0.63, 95% CI 0.52–0.74, for the two radiologists) was obtained, with no significant difference (*P* = 0.57 and *P* = 0.11, respectively) in comparison with MRI data (weighted *κ* = 0.73, 95% CI 0.62–0.84). In addition, substantial agreement was found between grades assigned using US and MRI (weighted *κ* = 0.61, 95% CI 0.50–0.72).

**Table 4:** Results of US and MR imaging for detecting knee cartilage defects of each grade

Grade	US		MRI
	Radiologist 1	Radiologist 2	
0	92.9 (78/84) (0.317)	90.5 (76/84) (0.083)	94.0 (79/84)
1	0.0 (0/20) (0.025)	0.0 (0/20) (0.025)	25.0 (5/20)
2	33.3 (10/30) (0.083)	40.0 (12/30) (0.317)	43.3 (13/30)
3	55.3 (21/38) (0.014)	65.8 (25/38) (0.157)	71.1 (27/38)
4	73.9 (17/23) (0.083)	82.6 (19/23) (0.317)	87.0 (20/23)

Data are the percentages, followed by the raw data; numbers in parentheses are the *P* values in comparison with MRI (McNemar test)

**Table 5:** Comparison of aberrations between US and MRI grades and surgical grades

	Modality	US radiologist 1	US radiologist 2	MRI
Undergrading	2–4 grades	29 (14.9)	14 (7.2)	19 (9.8)
	1 grade	18 (9.2)	12 (6.1)	14 (7.2)
Identical grading	–	126 (64.6)	132 (67.7)	144 (73.8)
Overgrading	1 grade	12 (6.2)	13 (6.7)	9 (4.6)
	2–4 grades	10 (5.1)	24 (12.3)	9 (4.6)

Data are the number of defects. Values in parentheses are percentages

### Reasons for false-positive and false-negative diagnoses

**Fig. 4:**
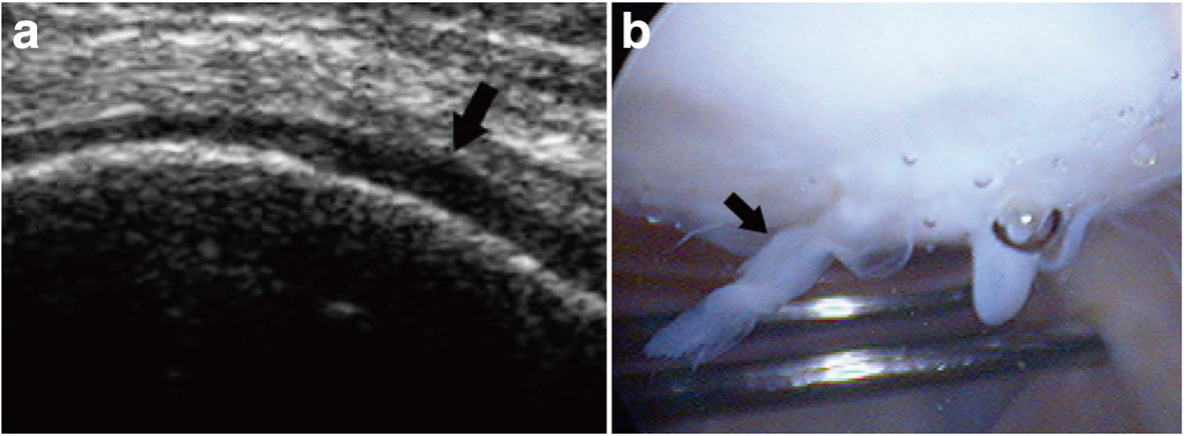
A grade 2 lesion at the lateral femoral condyles near the intercondyloid fossa, missed by US. **a** On the US, the diagnosis was normal (grade 0), but on arthroscopy **b**, the diagnosis was grade 2 (black arrow) cartilage defect, presenting as a velvet-like formation with intact cartilage surface, located at the lateral femoral condyles near the intercondyloid fossa

**Fig. 5:**
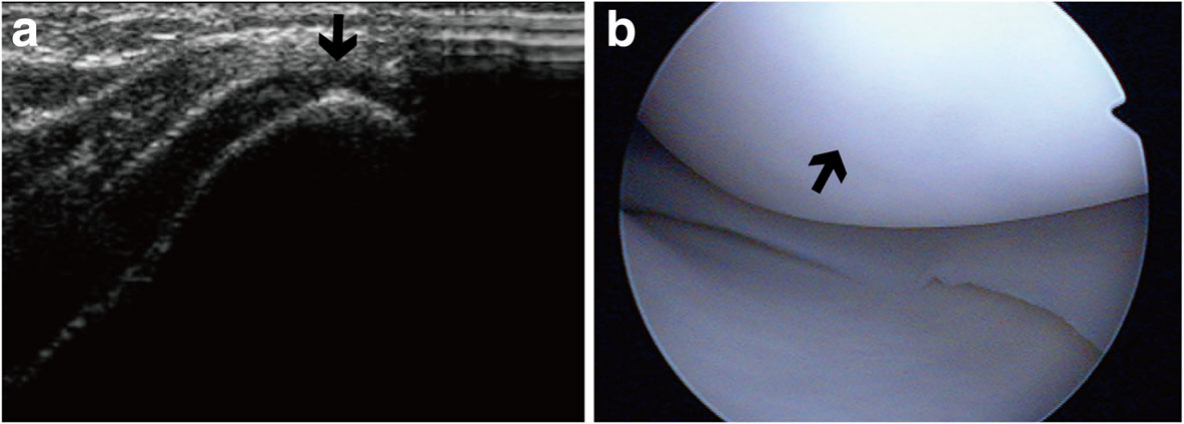
Example of a false-positive case. **a** The case was diagnosed as grade 3 cartilage defect on the trochlear surface (black arrow), presenting as blurred margin, lack of clarity, and overt local thinning (> 50% of cartilage depth). **b** On AS, the diagnosis was grade 0, i.e., normal cartilage (black arrow)

Error analysis was carried out to determine the reasons for false-positive and false-negative diagnoses by US, and the results are shown in Table 6. The majority of false negatives were due to the lesions being located at certain sites, including the femoral condyles near the intercondyloid fossa, where no defects were detected by US (Fig. 4). The majority of false positives were attributed to the partial volume effect (Fig. 5).

**Table 6:** Error analysis: reasons for false positive and false negative diagnoses

Surface	False-negative finding^#^			False-positive finding^#^	
	Particular sites	Partial volume effect	Small or superficial lesion	Thin cartilage	Partial volume effect
TS	0/0	3/3	4/3	0/0	2/3
MC	9/7	5/4	6/5	1/1	2/3
LC	11/9	4/3	0/0	0/0	1/1
Total	20/16	12/10	10/8	1/1	5/7

*TS* trochlear surface, *MC* medial condyles, *LC* lateral condyles

^#^Data are the surface numbers for radiologist 1/radiologist 2

## Discussion

In this study, we aimed to improve the diagnostic accuracy of US for the diagnosis of knee lesions with the intention of improving the accessibility and decreasing the costs of knee examination associated with MRI and arthroscopy, particularly for outpatients. Nevertheless, MRI and arthroscopy are still necessary. Therefore, this study assessed the diagnostic value of a novel US scanning approach in evaluating knee femoral cartilaginous defects and found that it allows a similar diagnostic performance as routine MRI, but with improved NPV compared with previous US scanning approach, which is of clinical significance. Different articular surfaces of the femur can be accessible with an external US probe by varying the angle range of knee flexion [23–25]. Interestingly, the novel scanning approach had similar sensitivity, specificity, and accuracy compared with MRI for detecting lesions on the whole femoral cartilage and individual articular surfaces. In addition, moderate agreement was obtained between grades assigned by arthroscopy and US.

In the present study, a substantial interobserver agreement was observed, while similar PPVs and higher NPVs were obtained in comparison with recent reports [13, 14]. The improved NPV (i.e., decreased false-negative rate) could be a consequence of more visibility of the femoral cartilage in the novel scanning approach. The fairly low NPV reported by Saarakkala et al. [14] is probably not related to an intrinsic limitation of the US itself or a need for higher resolution imaging but rather a lack of thorough observation of the overall femoral cartilage. An available acoustic window is the most important factor for US examination. The difficulty in visualizing the whole femoral cartilage due to the shadow of the patella and tibia is a major disadvantage. By using multiple knee angles, the novel approach markedly decreased false-negative femoral cartilage defect diagnoses.

In full extension (0°), the patella rests over the supratrochlear fat pad and is almost completely proximal to the superior border of the femoral articular cartilage [22, 23]. In this position, US can show the cartilage of the anterior and posterior femoral condyles but not the trochlear surface and weight-bearing femoral condyles due to the interference of the patella and tibia. In 10°–20° flexion, the patella first hugs the femoral shaft closely then slips distally and always remains in contact with the trochlear surface of the femur [22, 23], which may lead to poor visibility of most parts of the femoral articular surfaces. At approximately 135° of flexion, the patella reaches as far as the intercondylar notch [23]. At this time, good exposure of the entire trochlear surface and most parts of the lateral and medial condyles can be achieved. Therefore, scanning on minimum (0°) and maximum (≥ 135°) angles of the knee flexion may provide a more thorough scan of the femoral cartilage than those using only a fixed flexed knee (e.g., 120°).

MRI is considered a method of choice for thorough evaluation of cartilage morphology, but its routine use in all symptomatic patients with clinical suspicion of knee cartilage defects is limited due to unavailability in many district and community hospitals in China and high costs (in terms of money and time) [26]. Therefore, the application of the simple, widely available, and inexpensive US technique as the initial screening method for femoral cartilage lesions could be more appropriate. The novel US approach proposed here may satisfy the above requirements and can be used as an initial screening modality to provide a morphological assessment of the femoral articular cartilage in outpatient clinics.

As a non-invasive imaging modality, the novel approach needed further clinical validation. Therefore, the novel approach was compared with MRI, which is probably the most important method for cartilage imaging [26]. Previous comparative studies [27, 28] between US and MRI mainly focused on the thickness measurement of femoral cartilage, an important defect indicator, and showed a significant correlation (coefficients = 0.44–0.84). Comparison of US and MRI was further assessed in the present study; although a relatively lower detection rate of grade 1 defects was observed, the novel approach showed similar diagnostic ability for the detection and classification of cartilage defects compared with routine 2D FSE MRI, with a significant agreement for grading lesions.

The first major problem is that although the novel approach allows a significant decrease of false-negative diagnoses, it should be highlighted that the risk of false negatives was still as high as 26.7–30.5%, representing the majority of erroneous diagnoses. This likely results from the inability to visualize the lateral and medial condyles near the intercondylar notch, even at the maximum angle (e.g., 135°) of the knee flexion, due to their continuous articulation with the lateral and odd facet of the patella [15]. Thus, defects in these locations were the major cause of false negatives, as none of them was detected. Therefore, the blind areas of US have been improved by using varying flexion (0–135°) rather than a fixed flexion (120°), but the novel approach still needs to be improved. Patients suspected to be with cartilage defects should undergo additional diagnostic modalities, e.g., MRI, even with a negative US finding to verify the cartilage status.

The second major problem is that only femoral surfaces can be seen by US, not the patellar and tibial surfaces, which precludes the technique from providing an overall assessment of the knee articular cartilage. Nevertheless, strong correlations (Pearson’s correlation coefficients = 0.75–0.77) between volume changes in femoral cartilage and that in tibial cartilage in OA patients have been reported [29], indicating that evaluating one of the two features should be adequate. Therefore, US findings from the femoral cartilage might be reliable for evaluating arthritic cartilage changes of the tibial cartilage in clinical practice.

The US systems and technique are possible sources of error in US, as well as the operator. Although similar diagnostic accuracy between the novel approach and MRI was presented here, a substantial number of patients with small or superficial lesions (grade 1 defects) were misdiagnosed or missed by the US. Indeed, the US equipment available for routine clinical use can only assess conspicuous morphological changes of cartilage, not determining its internal characteristics, while MRI can. Therefore, subtle morphological changes in the early stage of cartilage defects might explain the misdiagnoses. More advanced equipment and techniques (e.g., a 50-MHz transducer, which can detect layers in immature cartilage [30]) may provide a solution. Further studies are necessary to verify this hypothesis. Another issue is operator dependency, a known problem in US examination [31]. As shown above, the difference in overall sensitivity was obtained between the two radiologists participating in this study. Therefore, a standardized training to learn the correct scanning approach and associated diagnoses is essential to avoid misdiagnoses or missed diagnoses.

There were some limitations to this study. First, although routine 2D FSE MRI sequences were performed as previously described [32–34], it may be argued that this study underestimated the actual diagnostic ability of MRI, as it is not optimal for cartilage evaluation due to anisotropic voxels, section gaps, and partial volume effects [26]. In addition, several MRI techniques are available to facilitate the assessment of the femoral cartilage for changes of morphology [35–37] and even biochemical composition [38, 39]. The results of MRI achieved in such sequences may be more favorable than those reported here. Nevertheless, since the cause of pain or disability of the knee is frequently multifactorial or unknown, 2D FSE sequences are most commonly applied in the clinical setting for initial examinations. In this study, we simulated a hypothetical situation of the first-time examination, which optimized the likelihood of screening cartilage defects.

Another limitation is that the same cartilage lesion could be attributed to different sites between US and MRI or arthroscopy. To minimize such discrepancies, the same standard schematic drawing of the femoral cartilage surfaces was used for all techniques. Nevertheless, a lesion located on the very edge of three articular surfaces would be likely assigned to different surfaces in various examination methods. Therefore, an overall assessment of the femoral cartilage from all three sites is necessary before the diagnosis and subsequent treatment of cartilage lesion; this is not affected by the possible misplacement. Further studies regarding treatment evaluation are required to target the precise lesion localization of cartilage.

In addition, the correlation between US findings and other assessment tools was not established. Indeed, in this initial study, we prioritized the associations of US with arthroscopy (gold standard) and MRI (most important imaging modality of cartilage). In the future, the correlation between the novel US scanning approach and clinical assessment should be evaluated for its recommendation in routine clinical use, including evaluation of degenerative changes and therapeutic effects.

The aim of the present study was to investigate the value of US as a screening tool for cartilage defects in patients with a chief complaint of knee pain (without any previous examination and diagnosis). It is indeed possible that some patients were not definitely diagnosed with OA. On the other hand, cartilage degeneration caused by OA may also present as cartilage defect. Therefore, it could be hypothesized that OA will not directly affect the capability of the US detection of cartilage defects, but this specific point will have to be examined in the future.

## Conclusions

The novel US scanning approach taking knee articular motion into consideration is more valid in a clinical setting to significantly decrease false-negative diagnoses compared with fixed-angle (120°) transverse scanning. It also has similar diagnostic performance, PPV, and agreement as routine MRI approaches for evaluating the knee cartilage defects in patients with a broad spectrum of knee diseases, but the NPV was higher than the previous US scanning approach. As a non-invasive, fast, inexpensive, and radiation-free imaging modality, US has a potential to be used for initial screening assessments of cartilage defects in first-visit patients with a chief complaint of knee pain and/or disability.

## Additional file

Additional file 1: **Table S1.** Parameters for MRI sequences. (DOCX 15 kb)

## Abbreviations

fs: Fat-saturated
LC: Lateral femoral condyles
MC: Medial femoral condyles
MRI: Magnetic resonance imaging
NPV: Negative predictive values
OA: Osteoarthritis
PD: Proton density
PPV: Positive predictive value
TS: Trochlear surface
US: Ultrasound
