# Supplementary material for: A novel ultrasound scanning approach for evaluating femoral cartilage defects of the knee: comparison with routine magnetic resonance imaging
Source: J Orthop Surg Res. 2018 Jul 16;13:178. doi: 10.1186/s13018-018-0887-x (PMC6048893; doi:10.1186/s13018-018-0887-x)
Supplement: Supplementary file 1 — Table S1. Parameters for MRI sequences. (DOCX 15 kb) [file 13018_2018_887_MOESM1_ESM.docx]

**Table S1** Parameters for MRI sequences

|  | MR Sequences | | | | |
| --- | --- | --- | --- | --- | --- |
| Imaging parameter | Sagittal T1 FSE | Sagittal T2 FSE | Sagittal fat-sat PD FSE | Coronal fat-sat PD FSE | Axial fat-sat PD FSE |
| Repetition time (ms) | 640 | 4400 | 2000 | 2000 | 2000 |
| Echo time (ms) | 8.6 | 82 | 27.8 | 27.7 | 27.7 |
| Field of view (cm) | 16 | 16 | 16 | 16 | 16 |
| Section thickness (mm) | 3 | 3 | 3 | 3 | 3 |
| Spacing (mm) | 1 | 1 | 1 | 1 | 1 |
| Echo train length | 3 | 19 | 8 | 8 | 8 |
